# Pre-radiotherapy ctDNA liquid biopsy for risk stratification of oligometastatic non-small cell lung cancer

**DOI:** 10.1038/s41698-023-00440-6

**Published:** 2023-10-02

**Authors:** Nicholas P. Semenkovich, Shahed N. Badiyan, Pamela P. Samson, Hayley B. Stowe, Yun E. Wang, Rachel Star, Siddhartha Devarakonda, Ramaswamy Govindan, Saiama N. Waqar, Clifford G. Robinson, Gregory Vlacich, Bruna Pellini, Aadel A. Chaudhuri

**Affiliations:** 1 Division of Endocrinology, Metabolism, and Lipid Research, Department of Medicine, Washington University School of Medicine, St. Louis, MO USA; 2 Department of Radiation Oncology, University of Texas Southwestern Medical Center, Dallas, TX USA; 3 Department of Radiation Oncology, Washington University School of Medicine, St. Louis, MO USA; 4 Siteman Cancer Center, Washington University School of Medicine, St. Louis, MO USA; 5 Tempus Labs Inc, Chicago, IL USA; 6 Division of Oncology, Department of Medicine, Washington University School of Medicine, St. Louis, MO USA; 7 Department of Thoracic Oncology, Moffitt Cancer Center and Research Institute, Tampa, FL USA; 8 Department of Oncologic Sciences, Morsani College of Medicine, University of South Florida, Tampa, FL USA; 9 Division of Biology and Biomedical Sciences, Washington University School of Medicine, St. Louis, MO USA; 10 Department of Genetics, Washington University School of Medicine, St. Louis, MO USA; 11 Department of Biomedical Engineering, Washington University in St. Louis, St. Louis, MO USA; 12 Department of Computer Science and Engineering, Washington University in St. Louis, St. Louis, MO USA

**Keywords:** Predictive markers, Non-small-cell lung cancer

## Abstract

The optimal treatment paradigm for patients with oligometastatic non-small cell lung cancer (NSCLC) remains unclear. Some patients with oligometastatic disease experience prolonged remission after locally consolidative radiation therapy (RT), while others harbor micrometastatic disease (below limits of detection by imaging) and benefit from systemic therapy. To risk-stratify and identify the patients most likely to benefit from locally consolidative RT, we performed a multi-institutional cohort study of 1487 patients with oligometastatic NSCLC undergoing liquid biopsy analysis of circulating tumor DNA (ctDNA). In total, 1880 liquid biopsies were performed and approximately 20% of patients (*n* = 309) had ctDNA measured prior to RT and after their diagnosis of oligometastatic disease. Patients with undetectable ctDNA (pathogenic or likely pathogenic variants in plasma using the Tempus xF assay) before RT had significantly improved progression-free survival (PFS) (*P* = 0.004) and overall survival (OS) (*P* = 0.030). ctDNA maximum variant allele frequency (VAF) pre-RT and ctDNA mutational burden pre-RT were both significantly inversely correlated with PFS (maximum VAF *P* = 0.008, mutational burden *P* = 0.003) and OS (maximum VAF *P* = 0.007, mutational burden *P* = 0.045). These findings were corroborated by multivariate Cox proportional hazards models that included eight additional clinical and genomic parameters. Overall, these data suggest that in patients with oligometastatic NSCLC, pre-RT ctDNA can potentially identify the patients most likely to benefit from locally consolidative RT and experience prolonged PFS and OS. Similarly, ctDNA may be useful to identify undiagnosed micrometastatic disease where it may be appropriate to prioritize systemic therapies.

Oligometastatic non-small cell lung cancer (NSCLC) offers a unique opportunity for personalized liquid biopsy-guided therapies. While NSCLC patients with widespread metastatic disease are incurable and generally have poor outcomes, patients with a limited metastatic burden of disease can sometimes achieve prolonged remission after definitive management of the primary tumor and metastatic sites through a combination of systemic agents and local consolidative therapies such as RT^1–3^.

However, identifying patients who have truly oligometastatic disease and are most likely to benefit from locally consolidative radiation therapy is challenging. Many patients with perceived oligometastatic NSCLC on imaging likely harbor undetected widespread metastatic disease (micrometastatic disease) below the limit of detection of current imaging technologies. Establishing a new liquid biopsy biomarker to segregate those patients with truly oligometastatic disease from those with widespread micrometastatic disease could alter treatment approaches. Patients with evidence of micrometastatic disease could be triaged to earlier systemic therapies or enrollment in clinical trials — in addition to sparing the costs, systemic therapy breaks, and potential side effects associated with local consolidative therapies. Similarly, clinicians could provide more concrete guidance to patients regarding the possibility of prolonged remission, and potentially offer more aggressive locally consolidative treatment in truly oligometastatic patients who do not harbor liquid biopsy evidence of micrometastatic disease.

We have previously shown that post-RT plasma circulating tumor DNA (ctDNA) is powerfully prognostic in localized NSCLC^4,5^. Here, we hypothesized that ctDNA analysis could be applied earlier (pre-RT) to risk-stratify patients with oligometastatic NSCLC and enable patient-personalized determination of local consolidative radiotherapy versus systemic therapy.

## Analysis of ctDNA

Median follow-up time after initial blood collection for liquid biopsy analysis was 10.3 months. Across all ctDNA assays, 3503 pathogenic or likely pathogenic variants were identified (1.8 variants/sample, mean). Of the sub-cohort of 309 patients who underwent liquid biopsy after the diagnosis of oligometastatic disease and before RT, 48% (*n* = 151) experienced progressive disease and 11% (*n* = 34) died during the study period. ctDNA quantitation was based on the detection of pathogenic or likely pathogenic variants in plasma. ctDNA was detected in 74% of oligometastatic NSCLC patients prior to RT (*n* = 230) while the remaining 26% (*n* = 79) had no detectable ctDNA pre-RT. Among the 230 ctDNA-detectable patients, 76% (*n* = 175) had 1–3 variants, while the remainder (*n* = 55) had ≥4 pathogenic or likely pathogenic variants present.

**Fig. 1:**
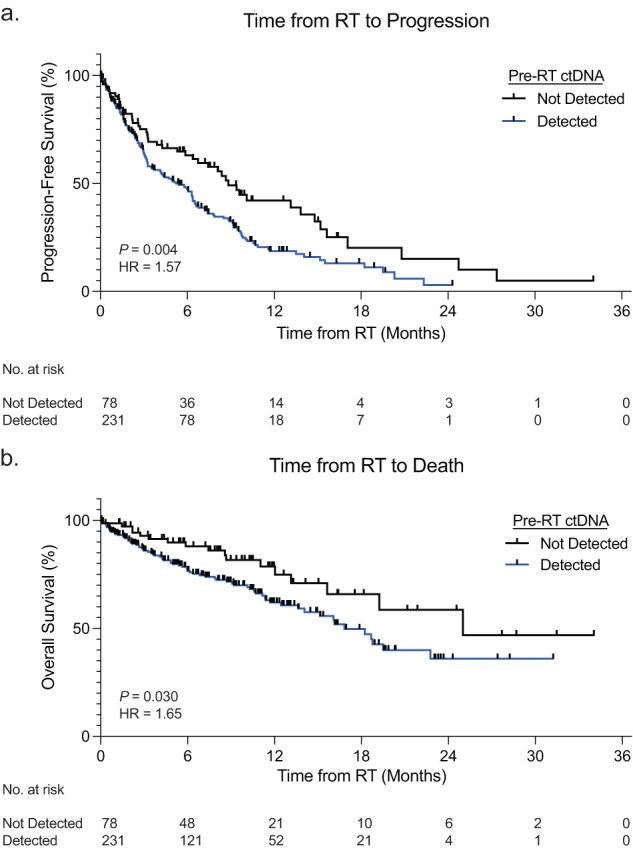
Survival stratified by pre-radiotherapy ctDNA detection in oligometastatic NSCLC. Kaplan–Meier curves demonstrating both progression-free survival (**a**) and overall survival (**b**) in oligometastatic NSCLC patients stratified by ctDNA detection prior to radiotherapy. *P* values were calculated by the log-rank test and HRs by the Mantel–Haenszel method.

Both progression-free survival (PFS) and overall survival (OS) were significantly worse in oligometastatic NSCLC patients with detectable ctDNA from pre-RT liquid biopsies, as compared to those without detectable ctDNA pre-RT. Patients with detectable ctDNA pre-RT had a median PFS of 5.4 months versus 8.8 months (*p* = 0.004, hazard ratio [HR] = 1.57, confidence interval [CI] = 1.15–2.13) [Fig. 1a]. Similar findings were observed for overall survival, with a median OS of 16.8 months versus 25 months (*p* = 0.030, HR = 1.65, CI = 1.05–2.61) [Fig. 1b].

**Fig. 2:**
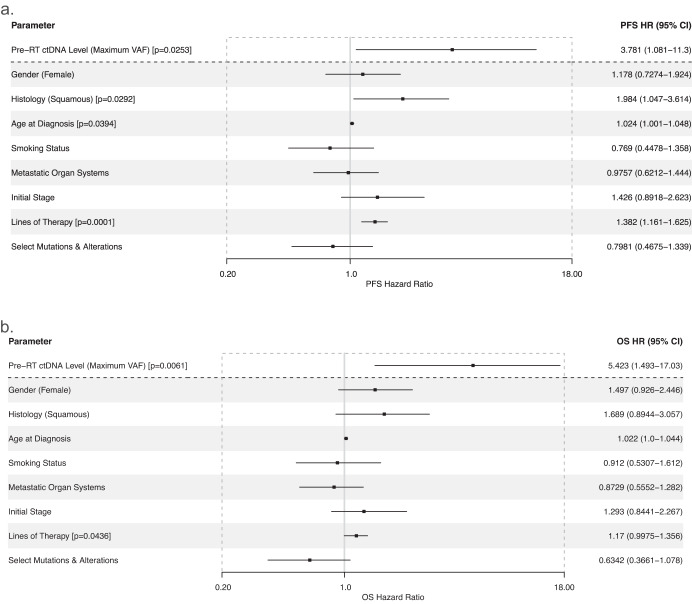
Multivariate Cox regression modeling of survival outcomes in oligometastatic NSCLC including the maximum ctDNA variant allele frequency. Multivariate Cox regression modeling was performed for (**a**) progression-free survival and (**b**) overall survival with parameters including the maximum ctDNA variant allele frequency (VAF) prior to radiotherapy, as well as clinically relevant covariates. Driver gene alterations include those defined in Tables 1 and 2.

ctDNA levels (defined by the variant allele frequency) demonstrated significant risk correlations, with the maximum pre-RT ctDNA VAF associated with increased risk of both disease progression (*p* = 0.008) and death (*p* = 0.007) [Supplementary Fig. 1]. These findings were corroborated by multivariate Cox proportional hazards models for PFS (*p* = 0.025, HR = 3.78, CI = 1.08–11.30) [Fig. 2a] and OS (*p* = 0.006, HR = 5.42, CI = 1.49–17.03) [Fig. 2b]. Notably, beyond pre-RT ctDNA levels, multivariate Cox modeling of OS only showed significant impacts from the lines of therapy a patient received (*p* = 0.044), while for PFS, squamous histology and age at diagnosis also demonstrated significance. Other clinical and genomic parameters including gender, smoking status, metastatic burden, initial disease stage, and presence of known common mutations and alterations were not significant with regard to survival outcomes.

Similar findings were observed when stratifying patients by pre-RT ctDNA mutational burden (the number of detectable pathogenic or likely pathogenic variants detected in plasma), with increasing ctDNA mutational burden associated with both progression (*p* = 0.003) and death (*p* = 0.045) [Supplementary Fig. 2]. These findings were again corroborated by multivariate Cox proportional hazards models for both PFS (*p* = 0.004, HR = 1.14, CI = 1.03–1.24) [Supplementary Fig. 3a] and OS (*p* = 0.014, HR = 1.13, CI = 1.02–1.23) [Supplementary Fig. 3b]. Beyond pre-RT ctDNA mutational burden, number of lines of therapy a patient received was again significant for OS on multivariate analysis (*p* = 0.039), while other clinical and genomic parameters were not.

## Translational implications

The definition of the oligometastatic disease state has remained frustratingly subjective since its original proposal in 1995 by Hellman and Weichselbaum^6^. Locally focused treatment for disease control was initially only considered in select NSCLC patients with a solitary metastasis in either the brain or adrenal gland^7^. More recently, phase 2 studies in patients with up to 3 or 5 metastatic lesions have shown improved survival outcomes when treated with locally ablative radiotherapy^1–3^. Current trials are now testing whether this radiation-oriented paradigm may also benefit patients with even greater numbers of metastatic lesions^2,8^, highlighting the need for more precise patient selection approaches. Our current work suggests that a pre-RT ctDNA liquid biopsy could serve as the first precision biomarker to objectively redefine oligometastatic disease, which would empower oncologists to provide more concrete advice to patients regarding RT for disease control and enable prioritization of systemic therapy for patients with ctDNA evidence of aggressive micrometastatic disease.

Indeed, to our knowledge, this study represents the largest ever real-world analysis of liquid biopsies in oligometastatic NSCLC, leveraging a multi-institutional dataset of 1487 patients who underwent 1880 liquid biopsies. Our analysis reveals that ctDNA testing performed pre-RT can risk-stratify those patients with truly oligometastatic NSCLC from those who likely harbor widespread micrometastatic disease (below current imaging limits of detection), a finding hinted at by other recent work^9,10^. Our modeling shows that the risk of disease progression and survival are informed by ctDNA quantitation, whether by mutational burden or the overall amount of ctDNA represented by VAF.

This approach should be prospectively evaluated in a clinical trial that redefines oligometastatic NSCLC to include a discrete liquid biopsy metric encompassing a low or undetectable ctDNA level. This technique may also be valuable for those patients who undergo curative-intent treatment for earlier stages of NSCLC, but subsequently develop oligometastatic disease (deemed “oligorecurrence”) and are weighing individualized treatment decisions.

### Limitations

This was a real-world study, with data collected from multiple clinical sites including both academic and community practices. Clinical data including metastases and RT were provided by the managing clinicians. Given incomplete data regarding the exact timing of oligometastatic disease diagnosis and RT, we explicitly focused on a sub-cohort where a liquid biopsy was definitively performed prior to RT administration in oligometastatic NSCLC patients [Supplementary Fig. 4]. Progression status was determined by individual clinician assessments and did not follow a standard criterion. Metastatic data were available at an organ system level and may not faithfully reflect disease volume. Notably, ctDNA levels did not correlate with metastatic burden [Supplementary Fig. 5]. This finding, combined with the fact that ctDNA correlated significantly with OS and PFS, suggests that ctDNA may more objectively reflect disease burden, aggressiveness, and underlying biology than classic imaging-based approaches to determining metastatic burden. Radiation therapy plans were determined by individual clinicians and did not necessarily target all metastatic sites. Although this introduces clinical heterogeneity into the dataset, it may also more accurately capture real-world clinical practice patterns and suggests broader extensibility of our clinical-correlative liquid biopsy findings. Moreover, our significant PFS data was corroborated by similarly significant OS data in this real-world cohort.

In conclusion, this study suggests that pre-RT ctDNA may be a powerful biomarker to accurately identify micrometastatic disease in patients with oligometastatic NSCLC. Earlier risk stratification using this liquid biopsy biomarker could support future clinical trials to enable personalized decision-making based on per-patient ctDNA risk profiles. Patients with high-risk ctDNA profiles could undergo systemic therapy prioritization and potentially escalation (avoiding systemic therapy breaks related to RT and potential RT toxicities), while patients with undetectable ctDNA or low ctDNA risk profiles could be offered locally consolidative stereotactic radiotherapy with biomarker-driven confidence.

## Methods

All analyses were performed using de-identified patient data. The study was exempt from institutional review board evaluation and informed consent given the de-identified nature of the data.

### Cohort selection

We leveraged a multi-institutional real-world cohort consisting of 1487 patients from both academic and community practices who were diagnosed with oligometastatic NSCLC. Peripheral blood samples were collected for liquid biopsy analysis between 2016 and 2022. The cohort mean age (SD) was 64.7 years (10.1), with similar numbers of male and female patients (784 female [53%], 703 male [47%]) [Table 1]. Approximately 73% of the patients had adenocarcinoma, 18% had squamous cell carcinoma, and 9% did not have histological subtyping available or had another subtype of NSCLC. Every patient underwent liquid biopsy and ctDNA analysis using the Tempus xF assay at different timepoints, for a total of 1880 ctDNA assays [Table 2]. All patients were reported by the treating physicians to have metastatic disease; we sub-selected a cohort for this analysis where a ctDNA liquid biopsy was definitively obtained after the diagnosis of oligometastatic disease but prior to radiotherapy (*n* = 309 patients) [Supplementary Fig. 4]. To control for selection bias, we performed a repeat analysis of all patients excluded from the sub-cohort (*n* = 1178) [Supplementary Fig. 6], which reassuringly demonstrated similarly significant findings of OS and PFS stratified by ctDNA results, however we chose to focus on the sub-cohort to avoid making assumptions about liquid biopsy timing.

**Table 1:** Cohort characteristics.

Characteristic	Entire Cohort (*n* = 1487)	Sub-cohort (*n* = 309)
Age, mean (SD), y	64.7 (10.1)	63.1 (10.1)
Sex		
Male	703 (47.3%)	149 (48.2%)
Female	784 (52.7%)	160 (51.8%)
Race and ethnicity		
Asian	93 (6.3%)	20 (6.5%)
Black	150 (10.1%)	38 (12.3%)
White	835 (56.1%)	176 (57.0%)
Other	44 (3.0%)	11 (3.5%)
Unknown	365 (24.5%)	64 (20.7%)
Primary Tumor Histology		
Adenocarcinoma	1078 (72.5%)	233 (75.4%)
Squamous	267 (18.0%)	50 (16.2%)
Other/Not Specified	142 (9.5%)	26 (8.4%)
Initial Reported Stage		
Not Reported	51 (3.4%)	1 (0.3%)
Stage 0	1 (0.1%)	0 (0.0%)
Stage I	87 (5.8%)	9 (2.9%)
Stage II	70 (4.7%)	4 (1.3%)
Stage III	272 (18.3%)	34 (11.0%)
Stage IV	1006 (67.7%)	261 (84.5%)
Driver Gene Alterations (Pre-Liquid Biopsy)		
*EGFR*	251 (16.9%)	53 (17.2%)
*ALK*	50 (3.4%)	9 (2.9%)
*ROS1*	21 (1.4%)	2 (0.6%)
*MET*	25 (1.7%)	8 (2.6%)
*RET*	7 (0.5%)	1 (0.3%)
*BRAF*	36 (2.4%)	9 (2.9%)
*KRAS*	72 (4.8%)	16 (5.2%)
*ERBB2*	14 (0.9%)	3 (1.0%)
Metastatic organ systems, mean^a^ (SD, range)	1.3 (0.64, 1–8)	1.3 (0.56, 1–5)
PD-L1 Status^b^		
Positive	302 (20.3%)	59 (19.1%)
Negative	202 (13.6%)	48 (15.5%)
Unknown	983 (66.1%)	202 (65.4%)
Smoking Status		
Current/Former Smoker	1006 (67.7%)	221 (71.5%)
Never Smoker	323 (21.7%)	73 (23.6%)
Unknown	158 (10.6%)	15 (4.9%)
Documented Radiotherapy	975 (66%)	309 (100%)
Reported Lines of Therapy		
Not Reported	212 (14.3%)	34 (11.0%)
1	674 (45.3%)	128 (41.4%)
2	374 (25.1%)	93 (30.1%)
3	141 (9.5%)	33 (10.7%)
≥4	86 (5.8%)	21 (6.8%)

The sub-cohort was defined as those patients who underwent a liquid biopsy ctDNA test prior to receiving radiation therapy and after their diagnosis of oligometastatic NSCLC. Data are presented as the number (percentage) of patients, unless otherwise noted. The mutations & alterations reported in this table reflect those reported by investigators and from paired tissue samples (results from cell-free DNA analysis are in Table 2).

^a^Metastases were reported at the organ system level. All patients were reported by investigators to have metastatic disease, but specific counts of metastatic organ systems were unavailable in 293 (19.7%) patients, excluding them from the sub-cohort.

^b^PD-L1 status was determined by values provided by the reporting clinician, or by tumor sample immunohistochemistry (IHC). For samples undergoing IHC, a tumor proportion score ≥1% was considered PD-L1 positive.

**Table 2:** Genomic alterations detected in cell-free DNA.

Characteristic	Entire Cohort	Sub-cohort
Liquid biopsies performed	1880	434
Variants detected (mean per ctDNA test)	38,546 (20.5)	9069 (20.9)
Pathogenic/likely pathogenic	3503 (9.1%)	828 (9.1%)
Conflicting evidence	17 (0.04%)	10 (0.11%)
Benign/likely benign	30,911 (80.2%)	7254 (80.0%)
Uncertain significance	4115 (10.7%)	977 (10.8%)
Driver gene alterations detected		
*EGFR*	94	15
*ALK*	9	1
*ROS1*	6	0
*MET*	11	4
*RET*	3	2
*BRAF*	11	0
*KRAS*	64	11
*ERBB2*	13	5

All patients underwent at least one ctDNA liquid biopsy test. Driver gene alterations encompass liquid biopsy detected SNVs, indels, fusions, and CNVs, and reflect only pathogenic or likely pathogenic findings.

### Liquid biopsy

The Tempus xF liquid biopsy assay is a laboratory developed test (LDT) designed to detect oncogenic and resistance mutations in cell-free DNA. The assay detects single nucleotide variants (SNVs), insertions/deletions (indels), rearrangements, copy number variations (CNVs), and microsatellite instability (MSI) with high sensitivity and specificity^11^. ctDNA results were analyzed for variants using VarDict^12^ and characterized as pathogenic or likely pathogenic based on their predicted functional impact as determined by SnpEff^13^ and clinical evidence, following the ACMG/AMP guidelines for variant classification^14^. We excluded variants considered benign, likely benign, or having conflicting evidence. Variants were quantified per patient, along with the maximum variant allele frequency (VAF). Fusions and CNVs were identified using SpeedSeq^15^ and CNVkit^16^, respectively. Patient-level data of time to outcomes, ctDNA mutational burden, VAF, and other parameters used in this study are provided in Supplementary Table 1.

### Statistical analysis

Median follow-up time was determined using the reverse Kaplan–Meier method. Outcomes for overall survival (OS) and progression-free survival (PFS) were both calculated from the start of RT (i.e., time from RT to death and time from RT to progression). Progression was reported by the managing clinicians. Kaplan–Meier curve *p*-values represent the log-rank test. Hazard ratios for OS and PFS in Kaplan–Meier analyses reflect the Mantel–Haenszel test. Multivariate Cox regression *p*-values were calculated using the Wald test. Data were managed using Apache Superset 2.1.0, and statistical analyses were performed using GraphPad Prism version 9.5.1 and R version 4.2.2.

### Reporting summary

Further information on research design is available in the Nature Research Reporting Summary linked to this article.

### Supplementary information

Supplementary Material

Reporting Summary

Supplementary Table 1

## Data Availability

The data supporting this study’s findings are within the article and supplemental files. Supplementary Table 1 contains deidentified patient-level data (including time to outcomes, ctDNA mutational burden, and other parameters) that can be used to reproduce the findings of this study.

## Code availability

The code necessary to perform statistical tests and reproduce the figures in this manuscript is publicly available at: https://github.com/semenko/oligometastatic-nsclc-2023
