# Supplementary material for: Pre-radiotherapy ctDNA liquid biopsy for risk stratification of oligometastatic non-small cell lung cancer
Source: NPJ Precis Oncol. 2023 Oct 2;7:100. doi: 10.1038/s41698-023-00440-6 (PMC10545784; doi:10.1038/s41698-023-00440-6)
Supplement: Supplementary file 1 — Supplementary Material [file 41698_2023_440_MOESM1_ESM.pdf]

Supplementary Figure 1: Survival stratified by pre-radiotherapy ctDNA levels in oligometastatic NSCLC

a.

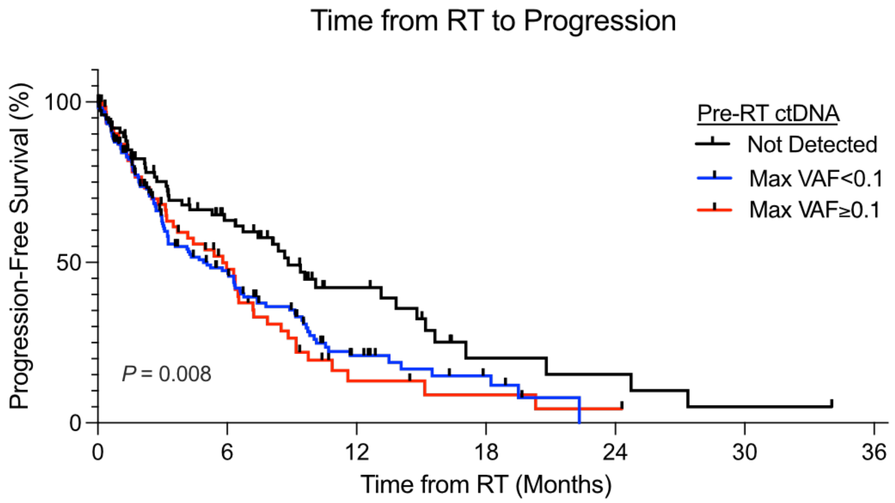

No. at risk

Not Detected	78	36	14	4	3	1	0
Max VAF <0.1	167	54	14	5	0	0	0
Max VAF ≥0.1	64	23	4	2	1	0	0

b.

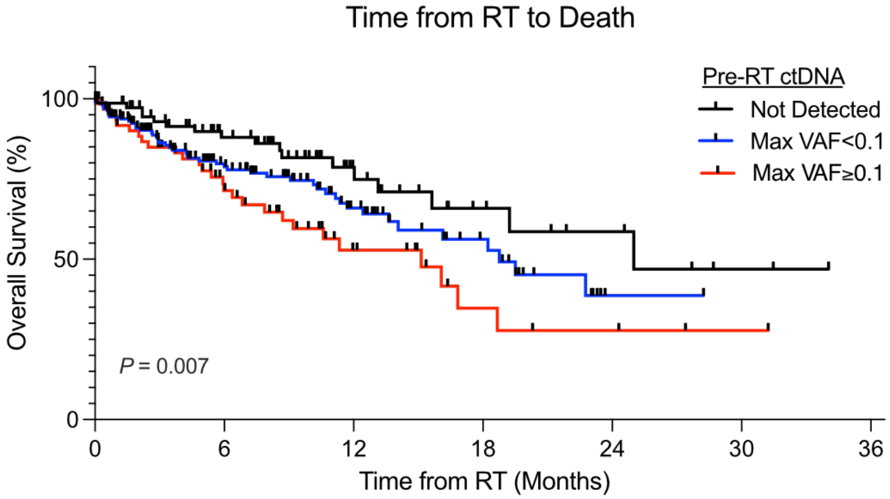

No. at risk

Not Detected	78	48	21	10	6	2	0
Max VAF <0.1	167	87	38	16	1	0	0
Max VAF ≥0.1	64	34	14	5	3	1	0

Kaplan-Meier curves demonstrating both progression-free survival (a) and overall survival (b) in oligometastatic NSCLC patients stratified by pre-radiotherapy ctDNA maximum variant allele frequency (VAF) levels. P values were calculated by the log-rank test for trend.

Supplementary Figure 2: Survival stratified by pre-radiotherapy ctDNA mutational burden in oligometastatic NSCLC

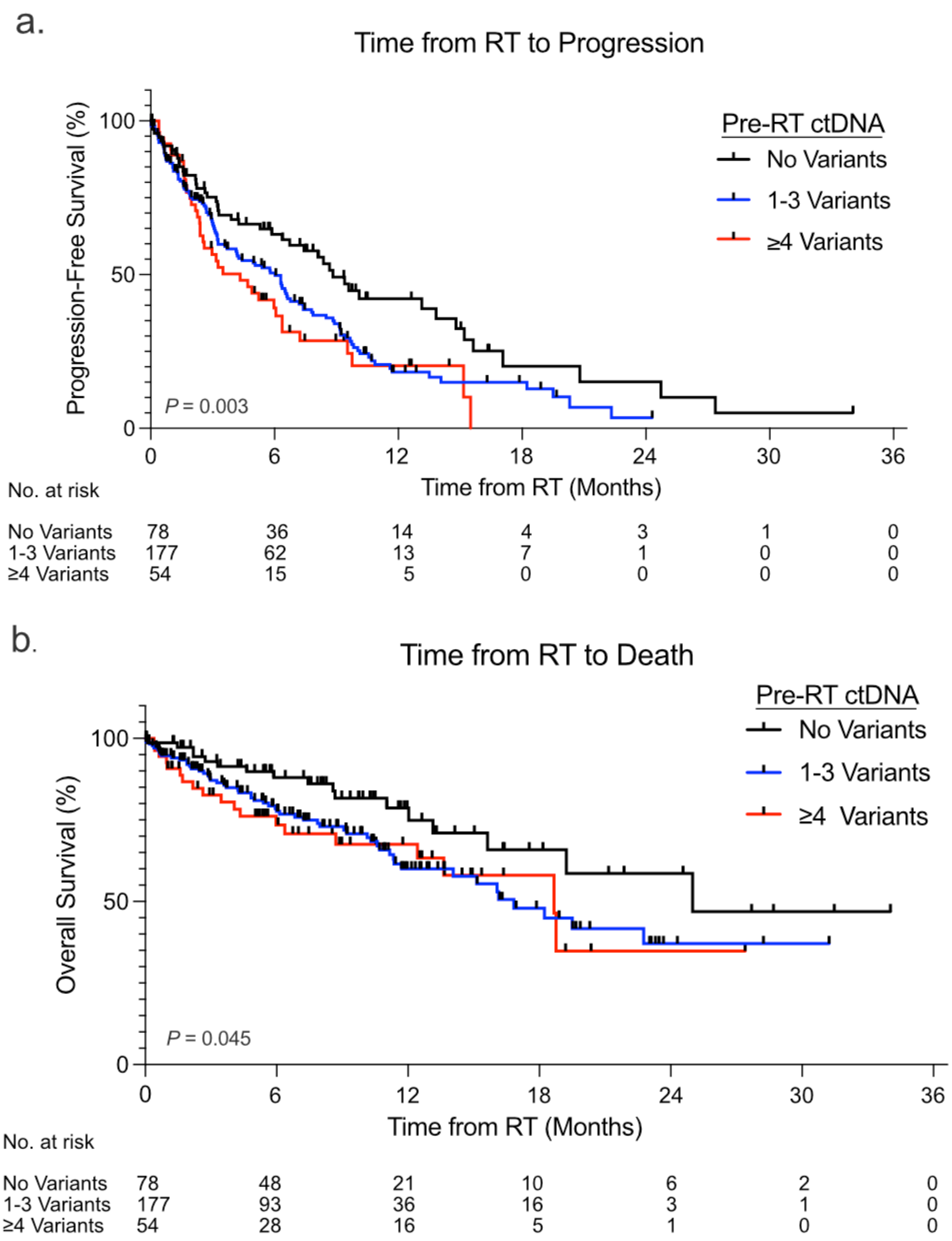

Kaplan-Meier curves demonstrating both progression-free survival (a) and overall survival (b) in oligometastatic NSCLC patients stratified by pre-radiotherapy ctDNA mutational burden. P values were calculated by the log-rank test for trend.

Supplementary Figure 3: Multivariate Cox regression modeling of survival outcomes in oligometastatic NSCLC including ctDNA mutational burden

a.

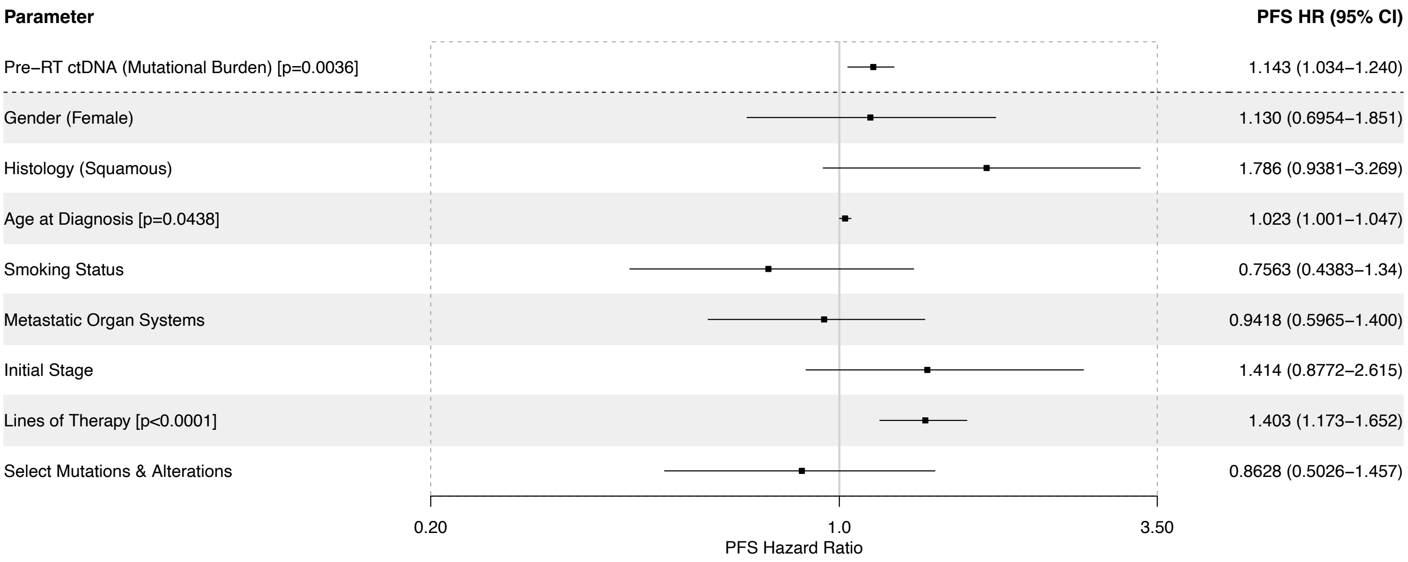

b.

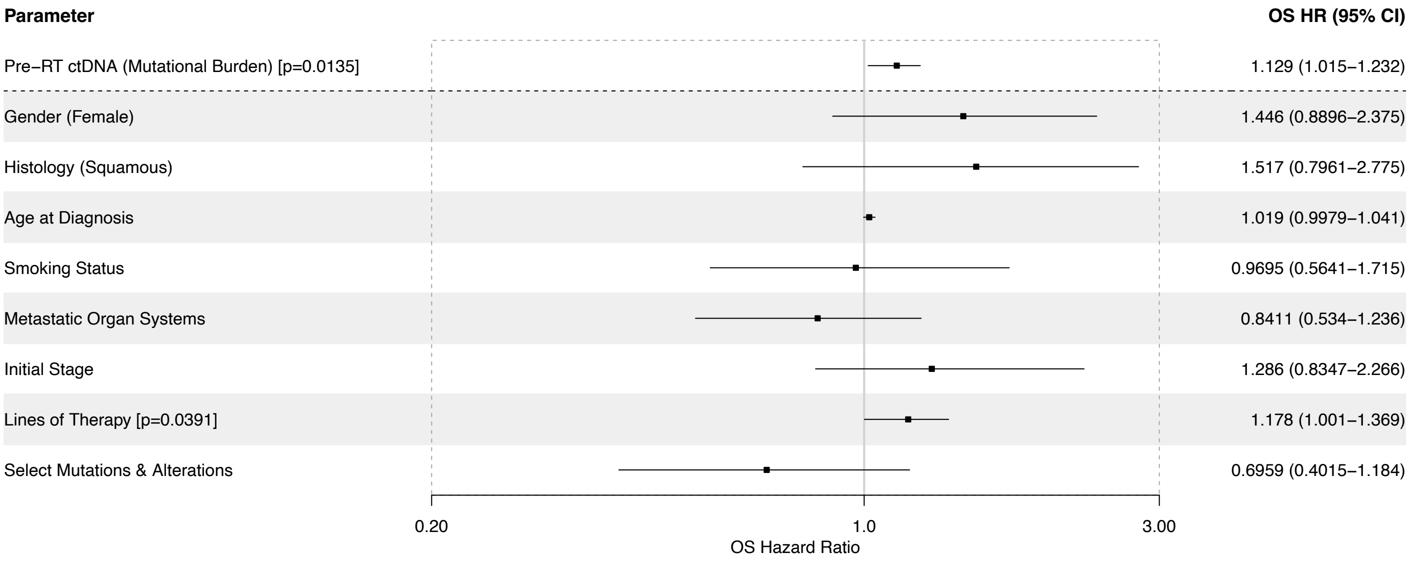

Multivariate Cox regression modeling was performed for (a) progression-free survival and (b) overall survival with parameters including the number of detected mutations in ctDNA (mutational burden) prior to radiotherapy, as well as clinically relevant covariates. Driver gene alterations include those defined in Tables 1 and 2.

Supplementary Figure 4: Diagram of sub-cohort selection

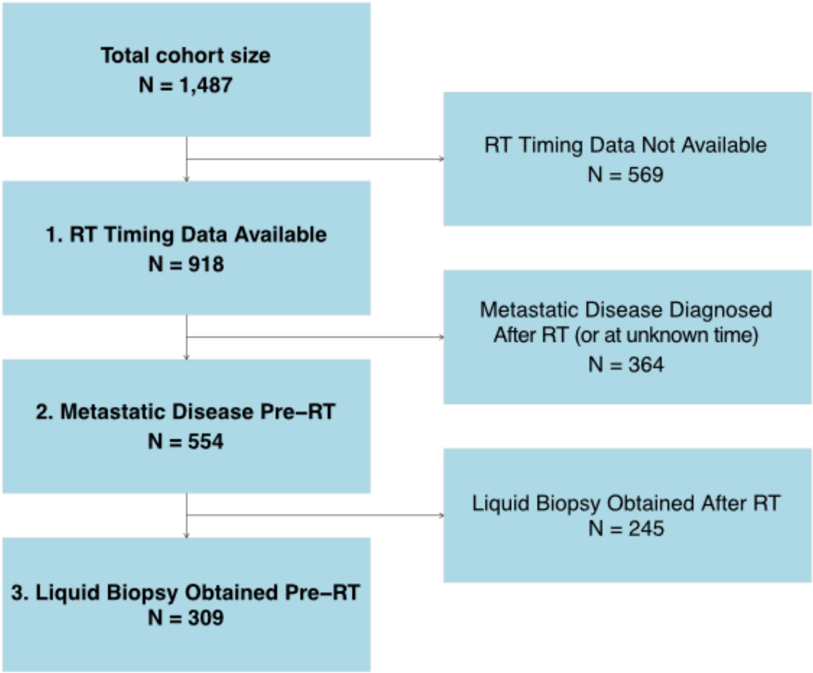

A sub-cohort was selected from the total patient population (n=1,487) based on the available clinical data. Patients were selected if: 1) data on the timing of RT was available, 2) data on the time metastatic disease was diagnosed was available, and metastatic disease was diagnosed prior to RT, and 3) liquid biopsy was performed prior to the initiation of RT.

Supplementary Figure 5: Correlations between ctDNA and metastatic burden

a.

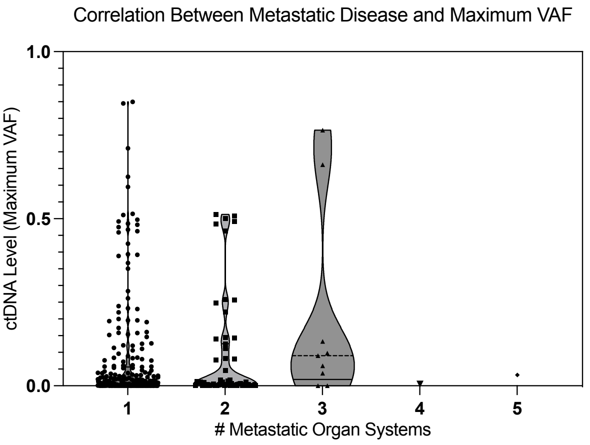

b.

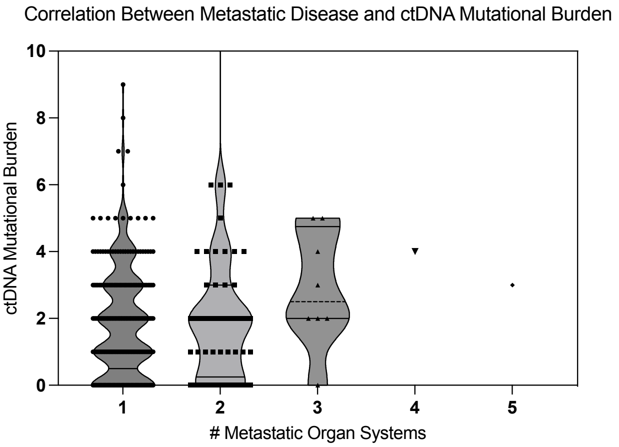

A correlative analysis was performed between pre-RT ctDNA and metastatic burden (represented by the number of metastatic organ systems). No significant correlation was identified between either a) the pre-RT ctDNA level as maximum VAF (one-way ANOVA P=0.16, R^2=0.015 by one-way ANOVA) or b) the pre-RT ctDNA mutational burden assayed by the number of pathogenic or likely pathogenic variants (P=0.32, R^2=0.015 by one-way ANOVA).

Supplementary Figure 6: Survival analysis of the non-selected cohort stratified by ctDNA detection in oligometastatic NSCLC

a.

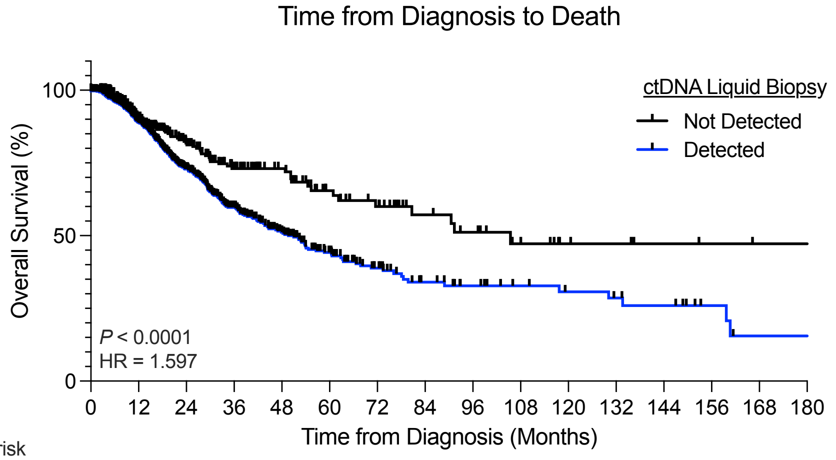

No. at risk

Not Detected	324	88	30	11	3	1
Detected	854	198	47	17	10	2

b.

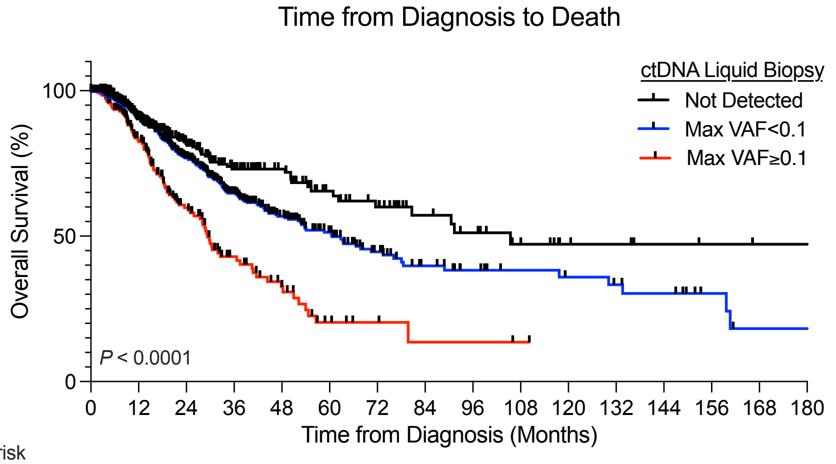

No. at risk

Not Detected	324	88	30	11	3	1
Max VAF <0.1	660	166	43	16	10	2
Max VAF ≥0.1	194	32	4	1	0	0

To evaluate for potential selection bias, we performed a survival analysis on the patients excluded from our sub-cohort (a total of n=1,178), where complete data on timing of RT and metastatic disease was not available. If multiple liquid biopsies were available for a given patient, we selected the earliest timepoint. Kaplan-Meier curves reassuringly demonstrated significant differences in overall survival in oligometastatic NSCLC patients stratified by (a) ctDNA detection and (b) ctDNA VAF levels. P values were calculated by the log-rank test and HR by the Mantel-Haenszel method.

Supplementary Table 1

oligometastatic_NSCLC_summarized_dataset.csv: Anonymized data corresponding to the sub-cohort (n=309) necessary to reproduce this work, including the ctDNA mutational burden, ctDNA variant allele frequency, time to progression, time to death, and clinical and demographic parameters.
