# Supplementary material for: Pre-radiotherapy ctDNA liquid biopsy for risk stratification of oligometastatic non-small cell lung cancer
Source: NPJ Precis Oncol. 2023 Oct 2;7:100. doi: 10.1038/s41698-023-00440-6 (PMC10545784; doi:10.1038/s41698-023-00440-6)
Supplement: Supplementary file 2 — Reporting Summary [file 41698_2023_440_MOESM2_ESM.pdf]

nature portfolio

Corresponding author(s): Aadel Chaudhuri, MD PhD

Last updated by author(s): Jun 7, 2023

Reporting Summary

Nature Portfolio wishes to improve the reproducibility of the work that we publish. This form provides structure for consistency and transparency in reporting. For further information on Nature Portfolio policies, see our Editorial Policies and the Editorial Policy Checklist.

Statistics

For all statistical analyses, confirm that the following items are present in the figure legend, table legend, main text, or Methods section.

n/a	Confirmed	
☐	☒	The exact sample size (*n*) for each experimental group/condition, given as a discrete number and unit of measurement
☐	☒	A statement on whether measurements were taken from distinct samples or whether the same sample was measured repeatedly
☐	☒	The statistical test(s) used AND whether they are one- or two-sided<br>*Only common tests should be described solely by name; describe more complex techniques in the Methods section.*
☐	☒	A description of all covariates tested
☐	☒	A description of any assumptions or corrections, such as tests of normality and adjustment for multiple comparisons
☐	☒	A full description of the statistical parameters including central tendency (e.g. means) or other basic estimates (e.g. regression coefficient) AND variation (e.g. standard deviation) or associated estimates of uncertainty (e.g. confidence intervals)
☐	☒	For null hypothesis testing, the test statistic (e.g. *F*, *t*, *r*) with confidence intervals, effect sizes, degrees of freedom and *P* value noted<br>*Give P values as exact values whenever suitable.*
☒	☐	For Bayesian analysis, information on the choice of priors and Markov chain Monte Carlo settings
☒	☐	For hierarchical and complex designs, identification of the appropriate level for tests and full reporting of outcomes
☒	☐	Estimates of effect sizes (e.g. Cohen's *d*, Pearson's *r*), indicating how they were calculated

Our web collection on statistics for biologists contains articles on many of the points above.

Software and code

Policy information about availability of computer code

Data collection	The data for liquid biopsies was collected and processed by Tempus Labs, with results generated by the xF analysis pipeline (V2.0, npj Precision Oncology PMID 34215841).
Data analysis	Data were analyzed using Apache Superset 2.1.0, R 4.2.2 (with packages forestplot 3.1.1 and dplyr 1.1.2), and GraphPad Prism 9.5.1. The code necessary to perform statistical tests and reproduce the figures in this manuscript is publicly available at: https://github.com/semenko/oligometastatic-nsclc-2023

For manuscripts utilizing custom algorithms or software that are central to the research but not yet described in published literature, software must be made available to editors and reviewers. We strongly encourage code deposition in a community repository (e.g. GitHub). See the Nature Portfolio guidelines for submitting code & software for further information.

Data

Policy information about availability of data

All manuscripts must include a data availability statement. This statement should provide the following information, where applicable:

- Accession codes, unique identifiers, or web links for publicly available datasets
- A description of any restrictions on data availability
- For clinical datasets or third party data, please ensure that the statement adheres to our policy

The data supporting this study's findings are within the article and supplemental files. Supplementary Table 1 contains deidentified patient-level data (including time to outcomes, ctDNA mutational burden, and other parameters) that can be used to reproduce the findings of this study.

Research involving human participants, their data, or biological material

Policy information about studies with human participants or human data. See also policy information about sex, gender (identity/presentation), and sexual orientation and race, ethnicity and racism.

Reporting on sex and gender	Sex is included as a clinical variable in our Cox models, and was based on investigator reported data (at the time liquid biopsies were submitted). In our analyzed subcohort, 149 (48.2%) were Male, and 160 (51.8%) were Female. Detailed cohort data is available in Table 1.
Reporting on race, ethnicity, or other socially relevant groupings	Race and ethnicity reporting was also included as a variable in our Cox models. Race and ethnicity values were based on investigator reported data at the time liquid biopsies were performed. Detailed race and ethnicity data are included in Table 1.
Population characteristics	Age, initial reported disease stage, and prior lines of therapy are all included in our Cox models to control for these covariates. These are also included both in Table 1 and in our supplementary data tables.
Recruitment	Participants were patients who underwent liquid biopsy testing from their individual clinician. Although this is becoming a standard of care, this may introduce biases in patients who did not have access to this test.
Ethics oversight	This study analyzed de-identified clinical data and was exempt from from institutional review board evaluation.

Note that full information on the approval of the study protocol must also be provided in the manuscript.

Field-specific reporting

Please select the one below that is the best fit for your research. If you are not sure, read the appropriate sections before making your selection.

☒ Life sciences ☐ Behavioural & social sciences ☐ Ecological, evolutionary & environmental sciences

For a reference copy of the document with all sections, see nature.com/documents/nr-reporting-summary-flat.pdf

Life sciences study design

All studies must disclose on these points even when the disclosure is negative.

Sample size	Sample size was based on available liquid biopsy data submitted for clinical processing, who had a reported diagnosis of metastatic NSCLC.
Data exclusions	We focused our analyses on a sub-cohort where all necessary parameters were available (including timing of radiation therapy, metastatic disease diagnosis, and liquid biopsy). We describe this selection in a CONSORT-style diagram (Figure S3) and performed an analysis of the excluded patients to ensure no bias was introduced by this sub-selection (Figure S5).
Replication	All attempts at replication were successful.
Randomization	This was a retrospective cohort study, no randomization was performed.
Blinding	Liquid biopsies were performed by individual investigators as part of their standard clinical workflow. Investigators had access to the liquid biopsy assay results to inform their clinical decision-making.

Reporting for specific materials, systems and methods

We require information from authors about some types of materials, experimental systems and methods used in many studies. Here, indicate whether each material, system or method listed is relevant to your study. If you are not sure if a list item applies to your research, read the appropriate section before selecting a response.

Materials & experimental systems

n/a	Involved in the study
☒	☐ Antibodies
☒	☐ Eukaryotic cell lines
☒	☐ Palaeontology and archaeology
☒	☐ Animals and other organisms
☐	☒ Clinical data
☒	☐ Dual use research of concern
☒	☐ Plants

Methods

n/a	Involved in the study
☒	☐ ChIP-seq
☒	☐ Flow cytometry
☒	☐ MRI-based neuroimaging

Clinical data

Policy information about clinical studies

All manuscripts should comply with the ICMJE guidelines for publication of clinical research and a completed CONSORT checklist must be included with all submissions.

Clinical trial registration	N/A, Not a clinical trial.
Study protocol	This study analyzed de-identified data submitted for liquid biopsies from patients with metastatic NSCLC. No standard protocol was followed by individual investigators.
Data collection	Liquid biopsies were collected by individual physicians as part of their standard clinical workflow.
Outcomes	The primary outcome was overall survival with a secondary outcome of progression-free survival. Progression events were determined by individual clinicians at part of their standard clinical workflow.
